# A JOURNEY INTO HOW PLACE MATTERS

**DOI:** 10.1093/geroni/igac059.555

**Published:** 2022-12-20

**Authors:** Terri Lewinson

**Affiliations:** The Dartmouth Institute for Health Policy and Clinical Practice, Lebanon, New Hampshire, United States

## Abstract

It is now well known that housing is a significant determinant of health and that systematic inequities shape the influence of home for many people. In this paper, I will present my research program designed to incrementally understand housing-health experiences of low-income residents in hotels, assisted living facilities, and senior housing apartments using qualitative, community-based, and biomedical approaches in Metropolitan Atlanta. Focused through an ecological lens, I will share insights from pedagogical and policy experiences that contributed to (and detracted from) the promotion of environmental justice for marginalized communities using inclusive strategies that were respectful of population identity. In this talk, I will discuss how institutional influences shaped opportunities for me to advance work on healthy housing as a social justice issue and detail my current project exploring air quality in a nontraditional home setting.

